# Product Diversity and Spectrum of Choice in Hospital ePrescribing Systems in England

**DOI:** 10.1371/journal.pone.0092516

**Published:** 2014-04-01

**Authors:** Hajar Mozaffar, Robin Williams, Kathrin Cresswell, Zoe Morison, Ann Slee, Aziz Sheikh Team, the ePrescribing Programme, Jamie Coleman, David W. Bates, Ann Robertson, Tony Avery, Laurence Blake, Antony Chuter, Sarah P. Slight, Alan Girling, Lisa Lee, Richard Lilford, Lucy McCloughan, Jill Schofield

**Affiliations:** Senior Clinical Lecturer, University of Birmingham; Professor of Medicine, Harvard Medical School; Research Fellow, The University of Edinburgh; Professor of Primary Health Care, The University of Nottingham; The University of Birmingham; Patient Representative; NIHR Career Development Fellow, The University of Nottingham; Senior Research Fellow, The University of Birmingham; Research Fellow, The University of Edinburgh; Professor of Clinical Epidemiology, The University of Birmingham; eHealth Research Manager, The University of Edinburgh; Head The York Management School, The University of York; 1 eHealth Research Group, Centre for Population Health Sciences, The University of Edinburgh, Edinburgh, United Kingdom; 2 Institute for the Study of Science, Technology and Innovation, The University of Edinburgh, Edinburgh, United Kingdom; 3 School of Health in Social Science, The University of Edinburgh, Edinburgh, United Kingdom; 4 Division of General Internal Medicine and Primary Care, Brigham and Women's Hospital, Harvard Medical School, Boston, Massachusetts, United States of America; National Cheng Kung University Medical College, Taiwan

## Abstract

**Background:**

ePrescribing systems have considerable potential for improving healthcare quality and safety. With growing expectations about the benefits of such systems, there is evidence of widespread plans to implement these systems in hospitals in England where hitherto they have had a low uptake. Given the international drive away from developing home-grown to systems to procuring commercial applications, we aimed to identify available ePrescribing systems in England and to use the findings to develop a taxonomy of the systems offered by suppliers.

**Methods and Findings:**

We undertook a scoping review of the published and grey literature, and conducted expert interviews with vendors, healthcare organisations and national ePrescribing experts in order to identify the spectrum of available systems, identify and map their key features, and then iteratively develop and validate a taxonomy of commercial ePrescribing systems available to English hospitals. There is a wide range of available systems including 13 hospital-wide applications and a range of specialty systems. These commercial applications can be grouped into four sub-categories: standalone systems, modules within integrated systems, functionalities spread over several modules, and specialty systems. The findings also reveal that apart from four packaged applications (two of which are specialty systems), all other systems have none or less than two live implementations across England.

**Conclusions:**

The wide range of products developed in the last few years by different national and international suppliers, and the low uptake of these products by English hospitals indicate that the English ePrescribing market is still in its infancy. This market is undergoing rapid cycles of change, both with respect to the number of suppliers and their diversity of offerings. Constant renewal of knowledge is needed on the status of this evolving market, encompassing the products development and adoption, to assist implementation decisions and facilitate market maturity.

## Introduction

The literature on hospital electronic prescribing (henceforth, ePrescribing) systems reveal the importance of these software applications in helping to enhance patient safety and to improve the quality and efficiency of healthcare [1]–[4]. The main demonstrated benefits of implementing ePrescribing systems include reduction of duplicate prescribing, dosing errors and issuing of and issuing of, contraindicated drugs, and enhancing adherence with formulary recommendations [2], [4]–[10]. With growing appreciation of these potential benefits, there is widespread plans to implement these systems into to hospitals in England where hitherto they have had little uptake [11]–[12].

Whilst earlier research on ePrescribing has focused on ‘home grown’ applications that have been internally developed by local teams [13], more recently the focus has shifted to implementing commercial of-the-shelf or ‘packaged’ ePrescribing systems [14]–[15]. These are diverse in terms of their functionality and complexity, ranging from basic data entry systems to more sophisticated applications providing medicines administration and decision support functionalities. Given the considerable work and resources involved in implementing these systems [16], it is therefore surprising that there is currently no comprehensive overview of the ePrescribing systems potentially available to National Health System (NHS) hospitals in England or indeed the key properties of these systems.

Technology suppliers have responded to growing expectations about ePrescribing benefits and policies encouraging their uptake by NHS hospitals. A wide and increasing range of providers are thus now offering their solutions to hospitals. Deciding on which system to procure is an important and costly decision with long-term consequences for the healthcare organisation concerned and possibly also for patient outcomes [16]–[17]. Given this situation, we sought to build on our national descriptive studies of planned implementations of ePrescribing systems [6], [12] and embarked upon a study of the current state of ePrescribing offerings available and, in some cases, in use in secondary care in England. Our earlier questionnaire study showed that while only 7% of English hospitals were using an ePrescribing system, 20% of hospitals were implementing and 55% were in the process of planning or procuring a system [6]. This work extends that study by aiming to provide an overview of the current ePrescribing market in England and develop a taxonomy of the systems offered by the increasing number of suppliers in this expanding market. We also sought to draw attention to the possible impacts of this diversity on decision making for uptake of ePrescribing applications.

## Materials and Methods

### Ethical Considerations

This study is a part of a national research programme investigating the implementation and adoption of ePrescribing systems in English hospitals, which received ethical approval from The University of Edinburgh's Research Ethics Committee. We also received guidance on 6 August 2012 from the NHS Health Research Authority NRES Committee London City and East that the study did not require review from an NHS ethics committee.

The data obtained for this work comprised of publicly available documents and participants who had given their written informed consent to participate and be interviewed. Interview data were anonymised for analysis.

### Overview of Research Methods

We used a mixed-method approach that involved discussion with experts, a scoping review of the literature and in-depth qualitative expert interviews. In order to help ground this work within the context of NHS England we used NHS Connecting for Health's working definition of ePrescribing systems [15]:

‘The utilisation of electronic systems to facilitate and enhance the communication of a prescription or medicine order, aiding the choice, administration and supply of a medicine through knowledge and decision support and providing a robust audit trail for the entire medicines use process’.

#### Data Generation and Analysis

Data generation and analysis for this study were performed concurrently in four stages as described below.

The first stage of data collection involved interviews with a network of recognised experts from NHS professional domains with the purpose of refining a baseline definition for the inclusion of applications as ePrescribing systems. This network consisted of members of NHS who had closely worked with and studied ePrescribing systems in England. In these interviews we asked the interviewees about what should be considered as ePrescribing system. The results were analysed in terms of UK terms for ePrescribing systems and hospitals. This resulted in the identification of search criteria for document searching shown in Table 1.

**Table 1:** Criteria for Web-Searches.

System	Market
Electronic Prescribing/E-Prescribing/ePrescribing/Electronic Prescription/E-Prescription/ePrescription	NHS/NHS Hospitals/NHS Trusts
Medication Administration	UK Secondary Care/English Secondary Care
HEPMA/EPMA	UK Hospitals/English Hospitals/UK Trusts/English Trusts
Prescribing Software/Prescribing Application	NHS Connecting for Health/NHS CFH
	UK User/UK Customer/UK Adopter/English User/English Customer/English Adopter

In the second stage of data collection, which gathered the main body of data for this study, we collected documents mainly from online sources and healthcare conferences. A summary of data sources types is shown in Table 2. In this stage, with the help of the research team and the study librarian we initially examined our internal programme library (collected by members of our research team over the last three years) for any documents previously collected from a range of healthcare conferences. These conferences were either those organised by our research team (such as ‘ePrescribing: everything you want to know but were afraid to ask! A symposium for the health service’ held in March 2013), or those attended by the programme team (such as ‘Electronic Prescribing in Hospitals: Moving Forward’ held on February 2012). Data sources from these conferences included suppliers' commercial materials, presentation slides, reports and papers publically distributed in conferences. This formed a provisional picture of the market, which was used as an initiating point for the document search process. In the document search process we used the search criteria (using a combination of keywords in column 1 and column 2 of Table 1 obtained from stage one) to find any online information available on suppliers of ePrescribing systems in England. This resulted in discovering four main sources of online documents for further examination: suppliers' commercial websites, NHS websites (including NHS hospitals and NHS Connecting for Health), academic journals, and online media (particularly E-Health Insider - EHI). These documents were analysed further to gather data on the state/progress of adoption of systems in English hospitals. This was done through examining data obtained from several data sources [18]. We started this by examination of the information available in each of these websites and their subordinate webpages. To do this we initially developed a table consisting of the suppliers and products details. Data for this table was initially generated from suppliers' websites. Then this data was checked against data obtained from NHS webpages and online media. This led to more in depth data for each of the suppliers/products in the list. It also led to discovery of some new ePrescribing products/suppliers in operation in England to be added to the list. These new data were once more checked with data from supplier websites. Hence a cyclic collection of data was carried out to identify the ePrescribing products in implementation and/or use in England. We compared our findings to the findings from the academic journals search (obtained using the same criteria as other online information sources) which did not lead to generation of any further findings. Through this examination of various data sources we obtained a more comprehensive and reflexive analysis of the data than would have otherwise been possible[18].

**Table 2:** Sources of Documentary Data^1^.

Type of document	Example Sources	Nature of data collected
**Conference Publications**	Publication on presentation slides from the ‘Electronic Prescribing in Hospitals - Moving Forward’ Conference, 28 February 2012, London. Conference website, ‘ePrescribing: Everything that you want to know but were afraid to ask!’, 27 March 2012, Birmingham, http://www.crfr.ac.uk/events/eprescribing/sponsors.html (Accessed 6, Nov. 2012)	Available products, status of adopter hospitals, products features and functionalities
**Commercial Material**	iSoft Electronic Prescribing and Medicine Administration Brochure. JAC Medicine Management Commercial Pack	Suppliers, products, adopter hospitals, products features and functionalities
**Supplier Websites**	http://www.alert-online.com. http://www.allscripts.com. http://www.ascribe.com. http://www.cambio.se. http://www.cerner.com. http://www.cis-healthcare.com. http://www.cse-healthcare.com. http://www.epic.com. http://www.isofthealth.com. http://www.jac-pharmacy.co.uk. http://home.meditech.com. http://www.medical.siemens.com. http://www.noemalife.com. http://www.systemc.com. http://www.tpp-uk.com.	Products, products structure, products features and functionalities, adopter hospitals
**NHS websites**	www.nhs.uk. www.england.nhs.uk. www.connectingforhealth.nhs.uk. http://data.gov.uk/publisher/nhs-connecting-for-health. http://www.srft.nhs.uk. http://www.lhch.nhs.uk. http://www.surreyandsussex.nhs.uk. http://www.changemodel.nhs.uk.	Strategies, status of ePrescribing in adopter hospitals
**Online Media**	http://www.ehi.co.uk. http://www.medicexchange.com. http://www.realwire.com. http://www.hoise.com. http://www.tmcnet.com. http://www.prnewswire.com. http://www.tmcnet.com.	Status of products in England, adopter hospitals

1 Websites were accessed several times from 1^st^ of November 2012 to 30^th^ of April 2013.

The overall findings included the current status of suppliers and implementing hospitals in England as well as systems that had once been implemented in hospitals, but had since been discontinued. We sought to examine discontinued products in order to have a more complete picture of the history of the market. Finally, in this stage we performed a further analysis of the data based on the form of solution and placement of ePrescribing functionality within the system to generate a taxonomy of products.

Then in stage three we performed qualitative interviews to attempt to identify any further data sources and to obtain a ‘respondent validation’ check [18], in terms of taxonomy of systems. In this manner, using purposive sampling we interviewed at least one supplier or adopter hospital from each of the types resulted from the stage two analysis. In this regard we did a ‘typical case sampling’ [19], in which we contacted various suppliers and user hospitals found in stage two. Our goal was to interview at least one supplier or user hospital in each of the categories to test our findings about the placement of the system in the defined category and if necessary to refine the categories. Participant characteristics are summarised in Table 3. The interviews were open and semi-structured with the interview guide focusing on three main points: (a) the nature of the ePrescribing product (this was the primary goal), (b) the trajectory of its growth (interviews with suppliers), and (c) strategies in procurement and implementation of the system (interviews with adopter hospitals). The interviews were anonymised and transcribed and read for checking the consistency of findings. For the purposes of this paper, we focused on responses to point (a) above on what the system covers (to find whether this is just an ePrescribing system or a wider hospital information system) and where the ePrescribing and medicine administration functionalities lay within the wider system. This was done through identification of the modules of each product and eliciting information on, where (in which module(s)) ‘prescription or medicine order’ and ‘administration and supply of a medicine’ (as initially identified in our definition of ePrescribing system, above) is taking place. We also analysed data on whether the system under examination is offering other hospital related functionalities (e.g. Pathology or Patient Administration System) in an integrated manner. The interviews also gave us further data on the diversity of different interpretations and understandings of ePrescribing systems. Finally in stage four, we contacted two recognised experts from NHS professional domains (previously interviewed in stages one), for a final validation of the findings. These individuals were selected based on criterion sampling technique [20] with the criteria of having experience in study of ePrescribing systems in England. The experts' inputs were used as the final validity check for the results.

**Table 3:** Participant Characteristics in Data Collection Activities.

Participant Number	Setting	Type of System
**1**	User	Type 2a System, Type 2d System
**2**	User	Type 2b System
**3**	User	Type 2c System
**4**	User	Type 1 System
**5**	Supplier	Type 2b System
**6**	Supplier	Type 2c System

As described above, data analysis was carried out simultaneously with data collection. We analysed the results from all data sources to create a summary of the current ePrescribing market in England. We then interrogated our results with the aim of categorising the products particularly in relation to the nature of the application and its integration with other hospital information systems. This was done by analysing the data through examination of how they define ePrescribing, the different available modules, and how the ePrescribing functionalities are distributed over the system. Finally we validated our findings through presenting our results to professional networks.

## Findings

We identified over 30 relevant websites, 14 documents collected from the programme library (including presentations slides and vendors' brochure), and undertook six in-depth expert interviews.

### ePrescribing Products Available in England

In analysing data collected from these sources, we found a diverse range of ePrescribing products and functionalities in use or available to English hospitals. Table 4 summarises our findings. Some systems have gone through changes of name, where the supplier name changed or where it was merged with other vendors or acquired over the years. The name in the ‘supplier name’ column shows the name of the supplier at the time of this research. The second column, ‘system’, indicates the name of the ePrescribing application as mentioned on vendors' websites. The third column provides a descriptive narrative of the vendor and the system extracted from publicly available, online documents. Column four, ‘Nature of the ePrescribing Functionality’, identifies the nature of the system functionality and its relation to wider systems where applicable. Column five identifies the date and country of company establishment. Finally, columns six and seven, indicate the progression of the product in the English market and provide some examples of customer hospitals adopting these systems. As can be seen in Table 4, we have provided a list of ‘example customers’ at different stages of the adoption process, ranging from the intention to implement to fully implemented and working cases and even discontinuation.

**Table 4:** ePrescribing Products in England.

Supplier^2^	System	Overall^3^	Nature of the ePrescribing Functionality^4^	Date and country of origin	Current Status in UK hospital market	Example Customers^5^
Alert Life Sciences Computing	ALERT Prescription	ALERT claims to offer a full medication monitoring cycle by connecting hospital and pharmacy services. The system offers workflows between physicians, nurses, ancillary staff, pharmacists and pharmacy technicians.	ePrescribing is offered as functionality within the Electronic Medical Record system.	1999, Portugal	Though market penetration hitherto has been limited, a number of implementations are in progress.	Brighton and Sussex University Hospitals NHS Trust (In progress^6^). Blackpool, Fylde and Wyre Hospitals NHS Foundation Trust.
Allscripts	Sunrise clinical manager (SCM)- Also known as Eclipsys	The Eclipsys (SCM) solution offers a wide array of acute care clinical information systems functions including the management of inpatient orders, documentation of all administered medications, and daily patient care interdisciplinary documentation.	ePrescribing is a separate module in an integrated clinical information system.	1995, US	Though market penetration hitherto has been limited, a number of implementations are in progress.	Liverpool Heart and Chest Hospital NHS Foundation Trust. Salford Royal NHS Foundation Trust (Moved from iClinical Manager [CSC] to SCM).
Ascribe	Ascribe ePMA	Ascribe ePMA aims to offer a range of functionalities including NHS specific practices such as independent prescribing, supplementary prescribing and patient group directions. The ePMA system is grown out of Ascribe's pharmacy system. The system has in-built medicines reconciliation and supports clinical screening by pharmacists. Ascribe's latest version is designed as a web-based product.	ePrescribing is a separate module in clinical system.	1984, UK	Though market penetration hitherto has been limited, a number of implementations are in progress.	Northern Lincolnshire and Goole Hospitals NHS Foundation Trust (In progress). East Lancashire Hospitals NHS Trust (In progress – live in two sites). The Christie NHS Foundation Trust (Cancer Centre- Chemotherapy Prescribing).
Cambio Healthcare Systems	COSMIC Medication	Cambio Healthcare Systems is one of the market leaders of healthcare information system solutions in Scandinavia. COSMIC is a suite of e-health applications that have been designed to support healthcare processes across a large geographic areas by different types of healthcare providers. The system can be implemented on a module by module basis integrated with existing legacy systems.	ePrescribing is a functionality within the Cambio- The Clinical 5 module of the PAS system.	1993, Sweden	At the time of this research, COSMIC is not being used in any English NHS hospitals.	Wrightington, Wigan and Leigh NHS Foundation Trust (contract awarded in 2010 but cancelled in 2012).
Cerner Corporation	Cerner ePrescribe	Cerner claims to be the largest provider of electronic medical systems in the United States. Cerner's features include medication review (inpatient prescribing and management, outpatient prescribing), private prescriptions, medication administration, pharmacy, and decision support.	ePrescribing is a separate module within the Cerner integrated solutions.	1979, US	Though market penetration hitherto has been limited, a number of implementations are in progress.	Newcastle upon Tyne Hospitals NHS (initially partnered with the University of Pittsburgh Medical Centre but are now supported by Cerner). Surrey and Sussex Healthcare NHS Trust (In Progress). Wirral University Hospital Trust (In progress). Croydon Health Services NHS Trust (In progress).
CIS Healthcare	Theriak	Theriak claims to support complete closed loop from prescribing to administration and bedside verification. Theriak offers solution for both general e-prescribing and complex chemotherapy prescribing. Theriak has a range of other features, including a control mechanism known as DAX (Drug Advice eXpert).	ePrescribing is offered as a separate system.	UK (date not available)	There are currently no live implementations of this product in England. There are two implementations which started in 2010 but were suspended in 2012.	Leeds Teaching Hospitals NHS Trust (Implementation started in 2010 and suspended in 2012). University College London Hospitals NHS Foundation Trust (Implementation started in 2010 and suspended in 2012).
CSC	LORENZO	Lorenzo is a web-based Electronic Health Record software introduced as part of the English National Programme for Information Technology. The whole functionality (consisting of an integrated hospital information system) is built whilst being implemented, with the intention to develop a system in collaboration with the NHS. ePrescribing functionality can be implemented once the organisation is using the Lorenzo Patient Administration System.	ePrescribing is a separate module within an integrated solution.	2012, UK	The initial stage of the Lorenzo e-prescribing solution has been deployed at one NHS Hospital in 2012. The NHS has announced that a further 20+ hospitals will receive this functionality and the ability to deploy the in-patient prescribing and medicines administration functionality when it becomes available towards the end of 2013.	University Hospitals Morecambe Bay (out-patient prescribing, medicines on admission and discharge prescribing with automatic medicines reconciliation).
CSC	MedChart ePMA	MedChart, offered originally by iSoft, which was then acquired by CSC, claims to supports end-to-end medication management from prescribing, medication monitoring, and dispensing through to administration. The solution incorporates clinical data from the patient's record and external databases to provide a coordinated approach to patient care.	ePrescribing is a separate module.	1996, Australia	MedChart has been implemented and is working in various NHS hospitals over the past year or so.	Stockport NHS Foundation Trust. Pennine Acute Hospitals NHS Trust. Harrogate and District NHS Foundation Trust. University Hospitals Leicester NHS Trust.
CSE	PICS	An in-house developed e-prescribing system for University Hospital Birmingham NHS Trust.	A standalone system with functionalities designed to match the exact need of each ward in a hospital.	1998, UK	This system is being used in one hospital. It started with one ward in 1999. The majority of the wards were live in 2006. The final ward went live in 2008. The system has not expanded beyond its developer hospital yet.	University Hospital Birmingham NHS Foundation Trust.
Epic	EpicCare	Epic is a large and growing EPRESCRIBING solution in US. The system installs with a pre-built Model System (including decision support, order sets, reports and documentation tools) and configures to meet specific workflow requirements.	ePrescribing is offered as functionality within different modules.	1979, US	Epic is new in the English market with implementation contracts in progress	Cambridge University Hospitals NHS Foundation Trust (In Progress). Papworth Hospital NHS Foundation Trust (In Progress).
IDX Systems Corporation (IDX) — Now a division of GE	LastWord	Consists of four older products: Flowcast (revenue cycle management for hospitals and physicians, including scheduling, billing, etc.), Groupcast (Financial management system for smaller provider groups), Carecast (integrated clinical and financial application for large hospitals- this included lastword system), and Imagecast (“filmless” radiology image workflow).	Data Not Available.	Data not available.	The system is no longer supported by the vendor. No implementations in progress in England.	Chelsea & Westminster Hospital NHS Foundation Trust (GE is not supporting the system - the NHS hospital has plans to move to a new system).
JAC	JAC ePrescribing	JAC provides pharmacy stock control, e-prescribing and medicines administration. JAC claims to include integrated in-patient and out-patient prescribing, discharge prescribing, decision support for providing warnings on allergies, drug-drug interactions and therapeutic duplicates, as well as bed-side medicines administration support and recording.	ePrescribing is offered as a separate system.	1983, UK	JAC has the largest number of live systems England.	Aintree University Hospitals NHS Foundation Trust. Alder Hey Children's NHS Foundation Trust. Basingstoke and North Hampshire NHS Foundation Trust. Heart of England NHS Foundation Trust. Chesterfield and North Derbyshire Royal Hospital NHS Trust. Doncaster and Bassetlaw Hospitals NHS Trust. Dorset County Hospital NHS Foundation Trust (Only discharge prescribing). Great Ormond Street Hospital for Children NHS Trust. Leicester Partnership NHS Trust. NHS Ayrshire & Arran. NHS Isle of Wight. Swindon & Marlborough NHS Trust (Only outpatient prescribing). Royal Cornwall Hospitals NHS Trust. Royal Liverpool and Broadgreen University Hospitals NHS Trust + Liverpool Heart and Chest Hospital NHS Trust. Sheffield Health and Social Care NHS Foundation Trust. Southampton University Hospitals NHS Trust. The Whittington Hospital NHS Trust. Winchester & Eastleigh Healthcare NHS Trust.
MEDITECH (Medical Information Technology, Inc.)	Magic (Old product in UK moving on to version 6)	MEDITECH supplies an integrated information system for various facilities in the health care industry. This enables health care providers to track a patient's history and/or monitor on-going treatment of chronic health problems.	ePrescribing functionalities are distributed within various integrated modules.	1969, US	MEDITECH has been in the English market for more than a decade with a number of Hospitals live on Magic. MEDITECH version 6 implementation in progress in hospitals.	Countess of Chester Hospital NHS Foundation Trust. City Hospitals Sunderland NHS Foundation Trust (rolled-out in 2006, moving on to new version in 2013). Burton Hospital NHS Trust (using old version, planning to move to new version in 2013). Rotherham NHS Foundation Trust (In Progress). Liverpool Women's Hospital NHS Trust.
NoemaLife	Galileo	Galileo offers different patient management processes, whether they are administrative or medical. This includes control of core facilities flows, care provision, administrative management of the patient, and integration of external documents.	ePrescribing is offered as a separate system.	1996, Italy	Galileo is not yet live in any NHS Hospitals but development is in progress.	Worcestershire Acute Hospitals NHS Trust (In Progress).
Siemens Health	Soarian Health Archive	Siemens Healthcare (formerly Siemens Medical Solutions, formerly Siemens Medical Systems, internally within Siemens known as “Med”) is a supplier to the healthcare industry. Soarian Health Archive is not an e-prescribing system, but has a range of prescribing functionalities.	ePrescribing functionalities distributed over several modules. No particular EPRESCRIBING module.	Germany (date not known)	This product has stopped being implemented.	Dudley Group NHS Foundation Trust (started implementing this product but then stopped).
System C	Medway	The Medway Electronic Patient Record (EPR) has built-in prescribing and medicines administration for both inpatients and outpatients. Medway EPRESCRIBING claims to offer comprehensive clinical decision support including information about drug-drug interactions, National Patient Safety Agency (NPSA) alerts, checks against pathology results. Full prescribing guidance can be accessed either through the First DataBank Multilex drugs knowledge base or locally-created content referenced within Medway.	ePrescribing is offered as a separate module integrated in the Medway EPR product family.	1983, UK	Medway is not yet live in any NHS Hospitals. But there are implementations in progress.	Staffordshire NHS Foundation Trust (In Progress). Royal Devon & Exeter NHS Foundation Trust (In Progress).
TPP	SystmOne	SystmOne claims to supports the NHS vision for a ‘one patient, one record’ model of healthcare. Professionals should be able to access a single source of information, detailing a patient's contact with the health service across a lifetime. The SystmOne E-Prescribing solution offers a complete inpatient and outpatient prescribing, administration and stock control solution for an acute setting.	The ePrescribing solution is offered as part of the SystmOne Acute module or can be deployed separately to integrate with an existing PAS system.	1997, UK	Though market penetration hitherto has been limited, a number of implementations are in progress.	Airedale NHS Foundation Trust (In Progress).

2 Data in this table is obtained from online sources or interview and updated as of the data collections dates indicated in the methods section. This table does not offer an evaluation of the systems or suppliers. It is only an indicator of their English market growth.

3 Information in this column is produced from data obtained from suppliers' online websites.

4 Information in this column is obtained through examination of suppliers online website contents.

5 This column shows some example customers at the time of this research.

6 ‘In progress’ column indicates that, based on our collected data, the ePrescribing system is not fully live yet. This could range from a signed contract to implementation stage.

Our findings show 17 different systems that have been available in the English market, of which two systems have been discontinued (and furthermore currently have no implementation in progress); one system's implementation has been suspended due to its supplier being placed in administration, and one live system has stopped being supported by its vendor. The remaining 13 have either live implementations, are in the process of being implemented, or have signed contracts with various hospitals. Some, such as Epic and Ascribe are new to the English market with a few implementations in progress while others, such as Cerner, JAC, and MEDITECH, are already live or are currently being implemented in a number of hospitals.

As can be seen from Table 4, not all of these systems were developed by UK suppliers. Indeed, only half of the products were designed and developed by UK based suppliers. The remaining half originated from various other countries including Australia, Germany, Italy, Portugal, Sweden, and the US. The majority of these systems were initially designed to cater for the need of the practices of their countries of origin and then expanded to the UK (and elsewhere). This diversity in the background of the products has resulted in a range of different pre-defined processes, tasks and workflows in the system.

In addition to the hospital-wide ePrescribing systems listed in Table 4, there are also a number of specialty products, particularly in chemotherapy, paediatrics and mental health in use in England and the wider UK. Amongst the most widely implemented specialty products is the ChemoCare system developed by CIS Oncology Limited in the UK. This system is the most widespread electronic chemotherapy prescribing software in use across over 60 NHS hospitals. Other examples of specialty systems include ARIA Oncology, a US system developed by Varian Medical Systems, Oncology Patient Management administration system (OPMAS) developed to meet the needs of chemotherapy, and RiO developed by CSE for mental health, learning difficulty and community services sector. The list is long, but as the aim of this study was to focus on hospital-wide ePrescribing systems (rather than specialty systems), we have not attempted to capture the entire range of specialty applications. We have only named a few to show appreciation of the existence of these products in the market.

### Diversity of Products and Taxonomy of Solutions

Our analysis of the current English market reveals a diversity of products, features and suppliers emerging over the past few years, but limited adoption of the majority of products. We can distinguish between systems in terms of available features. Most systems focus on a selected range of ePrescribing features and functionalities defined by the NHS [1]. The functionalities, which are said to be supported by the majority of systems are: in-patient prescribing in different areas of clinical speciality, out-patient prescribing, discharge prescribing, availability of connection with other hospital information system modules and applications – for example, stock control and ordering, varying levels of medication administration, different levels of medication screening for health professionals, different degrees of decision support capabilities, patient identification and grouping, and reporting features.

Apart from diversity of features and functionalities, the findings also point to a wide array of different ePrescribing ‘modes of supply’ (i.e. the way in which suppliers of technology make their products available to the market) and ‘forms of solutions’ (i.e. position of the ePrescribing systems in relation to other hospital information systems.).

By analysing these modes we offer a typology of ePrescribing systems into two overarching categories i.e. home-grown bespoke products and commercial packaged applications, with the latter category further divisible into four sub-categories: standalone systems, modules within an integrated system, functionalities spread over several modules, and specialty systems. While the main difference between bespoke and packaged applications is the mode of supply, the distinction between sub-categories within packaged applications is defined by the forms of the solutions.

In general, our findings show a wide diversity of systems ranging from those developed by enthusiasts within an NHS Hospital (there are many, we have only highlighted PICS in Birmingham), to “Commercial Off-The-Shelf” (COTS) systems bought from external suppliers to be configured and used within different hospitals. As can be seen from Table 5, COTS, also sometimes referred to as packaged applications, fall under different categories based on the nature of their integration and interoperability. Our results indicate a wide range of vendor offerings, but limited market adoption of the majority of products. In fact only one system has more than five live hospitals using the application. The majority of systems are having their first few customers either recently going live or still in the process of implementation. This suggests an immature, but potentially rapidly growing market. Moreover, unlike more mature applications such as enterprise resource planning systems [21], there is a lack of standard definitions and distribution of functionalities across and within systems. More precisely in the case of integrated systems, we can see that some applications have a separate module that covers the majority of features required for ePrescribing while other systems have these features distributed over several modules. This shows that a consistent definition of feature and their offerings and how these are to be supplied has not yet been achieved between the community of vendors and adopters.

**Table 5:** Typology of EPRESCRIBING systems.

System Type	Description	Example
**Type 1: Bespoke Systems**	Home-grown systems developed to meet the particular requirements of a single hospital. During the design process of these systems the user and vendor are directly connected and the aim is to solve the particular needs of the user organisation.	PICS
**Type 2: Packaged Application**	Applications designed with the aim of catering for the needs of various organisations. They tend to cover the ‘generic’ needs of adopter hospitals. Hence a standard system is designed to be implemented in different hospitals. These systems are configured (or parameterised) to meet particular needs of each hospital.	
Type 2a: **Standalone systems**	In this case the ePrescribing system is a separate application, not connected to wider hospital information system. Particular interfaces are required to link such applications to other systems.	JAC
Type 2b: **Modules within an integrated system**	A single module that performs various functionalities required for prescribing and administration of medicine and works as a part of a larger health information system. In such cases the ePrescribing module can be implemented as a whole unit at different points of time. Integrated systems tend to have more advanced decision support functionalities.	Cerner, Lorenzo
Type 2c: **Functionalities spread over several modules**	Similar to the above case, this type of system also integrates into a larger health information system. However it differs from 2b in that the ePrescribing functionalities are not compiled into a single module; rather they are performed by multiple integrated modules. Hence to have the entire functionalities, various modules must be implemented. Integrated systems tend to have more advanced decision support functionalities.	Magic, EpicCare
Type 2d: **Specialty systems**	These systems are designed to meet the particular needs of a specialty in health care. Similar to the above cases they are designed as standard packages, but with special features to cater for the particularities of that specialty care. The specialty systems may be designed as standalone applications or modules within an integrated system.	ChemoCare, ARIA Oncology

## Discussion

### Summary of the Main Findings

The ePrescribing market is still in its early stages in England; it is however rapidly expanding and changing in response to the increasing demand. There is evidence of a wide range of products entering (and sometimes leaving) the English NHS over recent years. These systems have been developed in various forms by different national and international suppliers. This diversity of choice has led to varied strategies in selection of the products in the secondary health sector in England. Hospitals need to make important decisions in relation to both the distributor and the choice of system. This recent rapid expansion in the UK market can to a large extent be explained by the recent demise of England's National Programme for Information Technology, which had resulted in central government contracting with a limited number of suppliers and had as a consequence led to non-preferred providers abandoning the UK market [22]. With such major recent fluctuations in the market, it is we believe important for NHS hospitals to have access to independent data on the range of available systems and insights into their functionality, interoperability potential and costs in order to inform the decision making process.

This work provides an overview of the current situation of ePrescribing applications in English hospitals and then categorises them into five different types in terms of modes of supply and forms of solution. In so doing, this study echoes the findings of our two earlier descriptive surveys of ePrescribing implementation in English hospitals [6], [12] by highlighting the move from adoption of bespoke applications to packaged systems.

### Implications of the Diversity on the Choice

The study shows that the choice between these categories has a number of potentially significant implications for the adopting hospitals. They include different degrees of integration and interoperability, variations in time and cost of implementation for adopting hospitals, and various degrees of alignment between the processes supported by products and hospital practices.

First of all there are important issues in need of attention around integration and interoperability of different applications. As explained earlier, ePrescribing systems maybe modules within other hospital information systems (such as Electronic Health Record - EHR systems), they maybe functionalities spread over several other modules, or they could be standalone applications with limited or no integration with other hospital information systems. Earlier research suggested that investing in increased integration is likely to be associated with fuller realisation of operational benefits [23]. Such benefits include synchronisation between hospital activities, integration of human resources and better efficiency and productivity. Integrated systems make access to information available at any place in the hospital. Instead lack of integration, can lead to multiple data entry points which can result in problems such as increased potential of error and greater operating costs.

Such integrations tend to be easier to achieve through integrated packaged applications rather than bespoke systems. As can be seen in the case of PICS (implementation started in 1998, the majority of wards live in 2006), the implementation time for bespoke applications tends to be much longer than packaged applications. It is something of a paradox that, due to frequent elaboration and alterations of requirements, addition of new functionalities and drift of the system and lack of adherence to standards and pre-defined practices, the implementation of bespoke systems tends to be more time consuming and require more effort than packaged systems [24]–[26]. As well as being time consuming, due to the need for in house development and maintenance teams, they are also more costly [27]–[29]. The costliness of such systems, has led to a move away from bespoke developments to adopting packaged applications.

Despite their benefits, packaged applications have faced a number of critiques by researchers. The main challenge widely discussed is the lack of supplier-user proximity [30]. This issue, which is in fact associated with all types of packaged systems leads to what some refer to as ‘misalignment’ or ‘misfit’ between a specific user organisation's needs and the standard application's generic functionalities [31]–[32]. This is in contrast to the offerings of bespoke systems in which the application is designed to meet the exact need of the individual organisation.

As noted above, packaged applications tend to be less costly and less time consuming than bespoke developments. Standalone applications also tend to be cheaper to procure and quicker to implement. These reasons have led to implementation of standalone systems by a larger number of hospitals. In this manner these hospitals have perhaps opted to achieve short term benefits while investing less in the product. Such standalone systems tend to be unconnected in nature and simple to configure [26]. However integration and interoperability of standalone solutions with wider systems may be costly and difficult to achieve following initial implementation [26]–[27].

A combination of more or less conflicting factors underpinned the diversity of choice in the English hospitals. They include different system functionalities and the extent to which they match the needs of English hospitals, differing degrees of integration and interoperability with other hospital information systems, cost of implementation, and time and effort required for adoption of the system.

### Considering the Findings in Light of Existing ePrescribing Market Studies

We have investigated the English ePrescribing market and revealed a wide range of offerings in England. To our knowledge, there are currently no similar studies that address the broad scope of ePrescribing systems in the English market. Our study, which confirms and extends our earlier findings from two questionnaire studies of the implementation and adoption of ePrescribing systems in English hospital [6], [12], may be the first to examine these systems and their market penetration.

A report by eHealth Initiative [33] suggests a model based on the sophistication of the system through what is described as ‘graduated levels’ of ePrescribing systems. The paper distinguishes very low level of ‘basic electronic references’ (Level 1 – i.e. systems with drug and formulary information but not used in prescribing), and moves up by defining ‘standalone prescription writers’ (Level 2 – i.e. creation of prescription with not long-term data about patients), systems with support of patient data (Level 3), systems with medication management (Level 4), systems with connectivity to other applications such as pharmacy (Level 5), and finally systems integrated with Electronic Medical Records (EMR – a subcategory of electronic health records) (Level 6). Sheikh et al. takes this model one step further and categorises these six levels into 3 types: standalone system (combination of levels 1 and 2), systems integrated with EHR (combination of levels 3, 4, and 5), and systems integrated with EMR (level 6). In doing so, they show that these integrations have a direct effect on the decision support functionalities and hence as we move up the levels, with higher integration, we are able to observe more advanced decision support functionalities. Further to this, the NHS recently published a report [34], which shows a similar view on the interoperability of various available systems. Our results confirm the existence of these levels of sophistication and interoperability with different hospital information systems, but we suggest that this categorisation is a partial view of the existing systems as it does not show different modes of supply and the potential of having the features spread over the wider integrated system. In this way our taxonomy offers a more comprehensive view of ePrescribing systems supply.

### Strengths and Limitations

As far as we are aware, this is the first in-depth empirical study of the English ePrescribing market. Furthermore, we are also not aware of any similar studies which present a detailed picture of the market in other countries. Our results are not only usable as a source of input to English and more broadly UK hospitals, but they can also be used as a basis for comparison to the market in other countries.

Out long-track of studying IT in healthcare, together with our prior work on ePrescribing in the NHS on which this study builds and our multidisciplinary knowledge and experience in the study of enterprise information systems in various sectors enabled us to relate our findings with the wider research agenda on information systems in organisations. This enabled us to draw on conclusions not only with respect to findings in health information system research but also to discuss the matters in light of the existing literature on other types of enterprise applications.

There are some limitations to this study, including a partial insight into the detailed functionalities and offerings of these highly complex software products compounded by incomplete public availability of information about products, reflecting commercial sensitivities. The properties of such highly complex software products cannot simply be verified by visual inspection, and only become fully apparent as they are implemented and used within specific organisational settings [26]. This limits the scope for detailed analysis and comparison of the features.

We used an online document search method followed by interviews in collecting the data for this study. This was done to show a snapshot of the English ePrescribing market and categorise the findings into meaningful groups. However due to the scoping nature of the searches and the limited number of expert interviews, care needs to be taken in interpreting our findings. Furthermore, as this work is grounded in the context of NHS England careful considerations are required to generalise these findings to non-UK settings and contexts.

A further difficulty was examination of a constantly changing market. As the aim of this research was to draw a picture of the current ePrescribing market in England, we tried to convey the most up to date findings as of the date of this research. However, product features and their adoption experience are both changing rapidly. This study needs to be updated continuously to offer a complete view on changes in the market.

## Conclusions and Ways Forward

The ePrescribing market in England is going through multiple cycles of change. Many new products are entering the market while existing technologies are undergoing rapid change. Moreover, there is no uniform conception of ePrescribing systems. Instead our observations showed a fluctuating definition, which is evolving as the technology and its usage are being shaped and reshaped. Hence, there is a double immaturity in the market, one in relation to the conception of the ePrescribing as a result of uncoordinated and sometimes inconsistent user needs and the other with regard to the uneven growth of the systems with differing modes of supply, form of solutions and functionalities. As the market becomes more mature, we would expect to see more standard definitions and interpretations of ePrescribing systems and their functionalities. Also, rather than limited adoption of a wide range of systems, we would expect to see higher rates of adoption of a limited number of ePrescribing systems. Such a market, with larger number of live implementations and more mature products, would facilitate decision making for procurement of desired products.

We present these mixed methods descriptive findings as a starting point for understanding the wide range of choices facing hospitals – between different ePrescribing categories and between different products within these categories – in order to facilitate better decision making. The wide range of available offerings, yet with few uptakes, reflects the immaturity of a market which is still undergoing rapid changes – a fact which will in turn necessitate frequent updates to the findings of this paper. Further work is also required to expand the current study in terms of increasing the level of detail regarding the functionality and features of the diverse range of applications. We also suggest that these parameters could be incorporated into appropriate assessment tools for assisting the process of selection of products.
