# Correction: Product Diversity and Spectrum of Choice in Hospital ePrescribing Systems in England

**DOI:** 10.1371/journal.pone.0103912

**Published:** 2014-07-23

**Authors:** The PLOS ONE Staff

There are two errors in the author byline in the XML version of the article. The sixth author’s name is spelled incorrectly. The correct name is: Aziz Sheikh. Jamie Coleman should not be listed as an author. The correct author byline is: Hajar Mozaffar, Robin Williams, Kathrin Cresswell, Zoe Morison, Ann Slee, Aziz Sheikh on behalf of the ePrescribing Programme Team.

## References

[1] Mozaffar H, Williams R, Cresswell K, Morison Z, Slee A, et al (2014) Product Diversity and Spectrum of Choice in Hospital ePrescribing Systems in England. PLoS ONE 9(4): e92516 doi:10.1371/journal.pone.0092516 24691517 10.1371/journal.pone.0092516 PMC3972198

